# Preparation and stability characterization of flavor ingredients in E-liquids for preclinical assessment of electronic nicotine delivering system products: a case study of 38 flavor ingredients in a single mixture

**DOI:** 10.1093/jat/bkaf031

**Published:** 2025-04-23

**Authors:** John H Miller, Thomas J Hurst, Niti H Shah, Jingjie Zhang, Felix Frauendorfer, Philippe Guy, Pierrick Diana, Anneke Glabasnia, Matteo Biasioli, Julia Hoeng, Davide Sciuscio, Patrick Vanscheeuwijck, K Monica Lee

**Affiliations:** Center for Research and Technology, Altria Client Services LLC, Richmond, VA 23219, United States; Center for Research and Technology, Altria Client Services LLC, Richmond, VA 23219, United States; Center for Research and Technology, Altria Client Services LLC, Richmond, VA 23219, United States; Center for Research and Technology, Altria Client Services LLC, Richmond, VA 23219, United States; Reseach and Development, Philip Morris International, Neuchâtel 2000, Switzerland; Reseach and Development, Philip Morris International, Neuchâtel 2000, Switzerland; Reseach and Development, Philip Morris International, Neuchâtel 2000, Switzerland; Reseach and Development, Philip Morris International, Neuchâtel 2000, Switzerland; Reseach and Development, Philip Morris International, Neuchâtel 2000, Switzerland; Reseach and Development, Philip Morris International, Neuchâtel 2000, Switzerland; Reseach and Development, Philip Morris International, Neuchâtel 2000, Switzerland; Reseach and Development, Philip Morris International, Neuchâtel 2000, Switzerland; Center for Research and Technology, Altria Client Services LLC, Richmond, VA 23219, United States

## Abstract

E-vapor products generate aerosols typically containing nicotine, flavor ingredients, and aerosol formers (propylene glycol and vegetable glycerine). While many flavor ingredients are ‘generally recognized as safe (GRAS)’ for oral use in the food industry, there exist knowledge gaps on their effects when delivered by the inhalation route. Due to the large number of available ingredients and potential combinations used to create e-liquids, toxicological and analytical evaluation of each flavor ingredient is impractical. Moreover, chemical characterization requires analytical methods to be developed and validated to measure key ingredients, as well as stability assessments to demonstrate that these test materials were stable during the testing period, which is equally challenging. In this study, we present a pragmatic approach of preparing ‘preblends’ prior to making a test formulation, containing 38 flavor ingredients as an example case, ahead of preclinical toxicity testing. We used the preblends to simplify the preparation and the characterization of test formulations, establishing the stability criteria for the subsequent toxicity testing. We prepared preblends by dividing the 38 flavor ingredients into five preblend groups based on structural moiety, solubility, and chemical reactivity. These preblends were mixed to make two different ‘final’ test formulations (containing all 38 flavor ingredients with and without nicotine). We evaluated the stability of the preblends and the two test formulations prior to the subsequent *in vivo* inhalation studies. Based on the analytical assessment, all the preblends were stable up to 4 weeks at 0°C–4°C. When all preblends were mixed, the test formulation was stable up to 3 days in the presence of nicotine and 10 days without nicotine when stored at 0°C–4°C. These stability results were used to set the frequency of preparing the test formulations for the *in vivo* inhalation studies, ensuring stability of test materials prior to the biological testing.

## Introduction

The health risk associated with the use of traditional tobacco products [1, 2] (such as conventional cigarettes) is well established [3], prompting the continued effort to develop potentially reduced risk products (PRRP) as alternative products for adults who smoke (AS) [4]. Inhalable PRRPs such as e-vapor products or electronic nicotine delivering systems may provide AS the desired nicotine by inhalation, preserving the rituals and sensory aspect of smoking but offer substantially reduced exposure to the toxic chemicals associated with tobacco combustion [5–9]. These smoke-free PRRPs collectively offer AS who are unable to or are unwilling to stop smoking, a reduced exposure alternative, potentially decreasing risks to smoking related diseases [7], thereby promoting public health.

In developing PRRPs, the role of flavor ingredients has been debated. While they contribute to taste and sensory experiences that may enhance satisfaction and switching away from cigarettes [10–12], toxicological profiles of many flavor ingredients and the mixtures of e-liquid formulations are not well understood. Many flavor ingredients are ‘generally recognized as safe (GRAS)’ for oral use in food [1, 2]; however, their toxicity potential via inhalation is not fully known. As part of product stewardship, flavor ingredients and their use levels should be toxicologically evaluated and when there are data gaps, *in silico, in vitro*, or *in vivo* studies [13, 14] could be considered as part of hazard characterization. At the same time, the large number of flavor ingredients and the vast number of possible combinations of mixtures in e-liquids present challenges [15]. For example, conducting standard *in vivo* toxicity testing would require years of testing and the use of thousands of laboratory animals. In addition, generating and characterizing e-vapor aerosols for inhalation exposures is technically challenging and currently without standardized characterization methods [16], making comparison of findings from different studies difficult [15]. Furthermore, to perform toxicological studies, test formulations need to be first prepared and characterized and their stability assessed for the intended exposure regimen and usage periods.

Recently, Sciuscio *et al*. [17, 18] presented a comprehensive framework on evaluating representative flavor ingredient mixtures for e-vapor products, utilizing structural grouping of ‘read-across’ [19, 20] or ‘flavor toolbox’ concepts. This approach proposes, instead of testing each individual flavor ingredient, their (worst-case or most likely toxic) flavor group representatives (FGRs) can be tested as mixtures for inhalation studies. Prior to conducting *in vivo* inhalation testing of the prototype test mixtures, Sciuscio *et al*. proposed to first ensure the test formulation is prepared according to its stability and for the intended testing period [21] as part of test article characterization [22–27]. Accordingly, this study is part of the framework presented in Sciuscio *et al*. in that we prepared and characterized the test formulations, before the *in vivo* (up to 6 h/day) inhalation exposures.

The goals of this work were to present a pragmatic approach of using preblends for 38 flavor ingredients that simplify the preparation of test formulations and establish the stability criteria for the subsequent toxicity testing. Herein, we describe how we prepare the preblends for the test formulations (i.e. 38 FGRs up to 18% flavor loads in e-liquid formulations) [17, 18]. By preparing the concentrated premixtures (referred to as preblends) before making the test formulation, we are able to maximize stability and simplifying the preparation for the daily inhalation exposures. Although our approach is developed for e-vapor toxicity testing, the concept of concentrated preformulations is commonly used in both the food and beverage industries to improve stability [28].

## Materials and methods

### Preparation of preblends and test formulations

**Figure 1.**
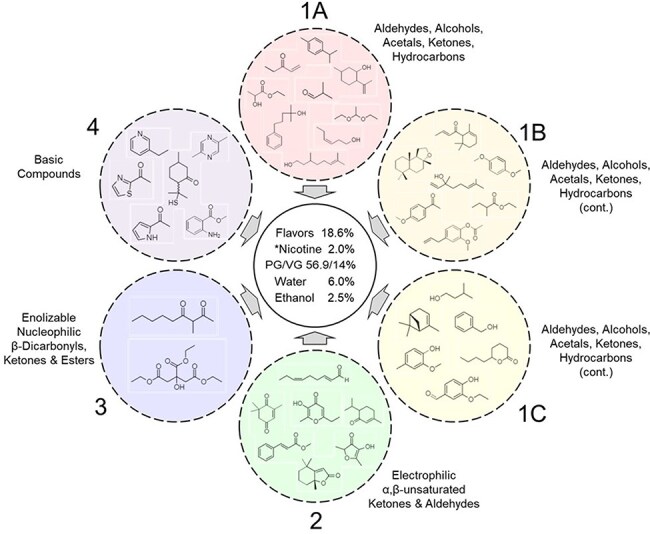
Structural groupings of preblend categories.

Sciuscio *et al*. [17, 18] selected 38 FGRs based on the read-across concept on similarity in their structural, toxicological, and metabolic properties. In this study, the 38 FGRs were analytically grouped into five chemically distinct subcategories (preblends, Table 1) based on the chemical characteristics as follows: first, comparable functional group reactivity, with alcohols being the least reactive and acids/bases being the most reactive (alcohols < aldehydes, ketones, and esters < enolizable aldehydes and ketones < α, β-unsaturated ketones and aldehydes < acids and bases); second, according to the reactivity between preblends to minimize impact on stability of the mixtures, to reduce the frequency of preparing the test formulations. Accordingly, a total of five preblends were prepared as summarized in Table 1. Due to the high number of individual ingredients, the Preblend Group 1 was further divided into three subgroups (1A, 1B, 1C) based on concentration and volatility (Fig. 1).

**Table 1.** List of 38 flavors in preblends [18]

Preblend	Compound ID	CAS No.	Preblend composition % (w/w)
Group 1A	Propylene glycol	57-55-6	60.894
	Paracymene	99-87-6	0.258
	1-Penten-3-one	1629-58-9	1.036
	Isopulegol	89-79-2	3,626
	Isobutyraldehyde	78-84-2	3.626
	d-l-citronellol	106-22-9	3.626
	Ethyl lactate	97-64-3	3.626
	*cis*-3-Hexenol	928-96-1	5.180
	Acetal	105-57-7	7.770
	2-Methyl-4-phenyl-2-butanol	103-05-9	10.358
Group 1B	Linalool	78-70-6	5.179
	Ethanol	64-17-5	6.250
	Ambrox (Cetalox^©^)	3738-00-9	2.590
	*para*-Dimethoxybenzene	150-78-7	2.590
	Propylene glycol	57-55-6	55.163
	*trans*-α-Damascone	24720-09-0	3.108
	Ethyl 2-methylbutyrate	7452-79-1	7.769
	Acetanisole	100-06-1	7.769
	Eugenyl acetate	93-28-7	9.582
Group 1C	Ethanol	64-17-5	6.250
	Alpha-pinene	80-56-8	6.474
	Delta-nonalactone	3301-94-8	5.180
	Isoamyl alcohol	123-51-3	6.474
	Benzyl alcohol	100-51-6	6.474
	2-Methoxy-4-methylphenol	93-51-6	6.474
	Ethyl vanillin	121-32-4	6.474
	Propylene glycol	57-55-6	56.201
Group 2	Ethanol	64-17-5	6.250
	Methyl cinnamate	103-26-4	2.590
	Propylene glycol	57-55-6	59.178
	Dihydroactinidiolide	15356-74-8	0.259
	Furaneol	3658-77-3	1.424
	Ethyl maltol	4940-11-8	12.948
	(E, Z)-2,6-Nonadienal	557-48-2	1.813
	Piperitone	89-81-6	12.948
	Ketoisophorone	1125-21-9	2.590
Group 3	Propylene glycol	57-55-6	96.140
	3-Methyl-2,4-nonanedione	113486-29-6	0.234
	Triethyl citrate	77-93-0	3.626
Group 4	Propylene glycol	57-55-6	72.314
	2-Acetylpyrrole	1072-83-9	1.036
	2-Acetylthiazole	24295-03-2	0.234
	3-Ethylpyridine	536-78-7	2.072
	*para*-Mentha-8-thiol-3-one	38462-22-5	3.626
	Methyl anthranilate	134-20-3	3.626
	2,5-Dimethylpyrazine	123-32-0	17.092
Group 5	2-Methylbutyric acid	116-53-0	100.000

For the preparation of the preblends, individual flavor ingredients (Table 1) were added in an amber coloured glass bottle, to protect the samples from light, in a sequential order to ensure solubility and then thoroughly mixed. For the preparation of the test formulations, each preblend was added to an amber glass bottle in the sequential order (Fig. 1). The last ingredient, 2-methylbutyric acid (0.2% w/w), was added to the mixture after adding all four preblend groups. For the test formulation without nicotine, propylene glycol (PG)/ vegetable glycerine (VG) (4:1 ratio) was used to offset the missing weight of nicotine. Identical amounts of water (5.9% w/w) were added to each formulation and thoroughly mixed using a magnetic stir bar on a stir plate. To reduce the volatility of flavor ingredients in the headspace (HS) and to minimize oxidation, preblend and test formulation mixtures were filled to the maximum level (∼95%) of an amber vial, capped and wrapped with parafilm for single use only (i.e. once the vial was opened and analyzed at a specific time point, it was discarded). After aliquoting, test formulations were stored in refrigerated or room temperature conditions.

### Stability study design

Preblends and test formulations were analytical characterized to establish the stability criteria for the toxicity testing period and to determine the preparation schedule. The stability for the preblends was determined over a 4-week (30 days) period, while stability for the test formulations was assessed over a 10-day period, each under room temperature and refrigerated storage conditions. We evaluated test formulations with and without nicotine (2%) to assess whether the addition of nicotine impacts the stability of flavor ingredients. Prior to analysis, samples were equilibrated to room temperature and vortexed thoroughly. We established the stability acceptance criteria for the preblends and the test formulations in that the compounds in the test articles were within the acceptable level for the study as follow: acceptance criteria were ±15% of the initial value (*T*_0_) for each preblend and ±20% of the initial value (*T*_0_) for the test formulations. The stability time was set at the point the measured values from two consecutive time points fell outside the acceptable range. Additionally, any unexpected peaks found in the total ion chromatograms (TICs) were further investigated to determine molecular weight/formula.

### Analytical characterization of preblends

Additional information related to the instrumental parameters used for the analysis can be found in the Supplemental Data. For Preblends 1A and 1B, an intermediate solution was made by diluting approximately 20 mg of the preblend with dichloromethane (DCM) and methanol (MeOH) (80:20 v/v) to a final concentration of 5 mg/mL. Internal standard solution (isophorone-d_8_) at a concentration of 0.2 µg/mL was added to the intermediate solution. Samples were further diluted between 20- and 250-fold to achieve an appropriate analyte concentration within the linear range of the instrument. These samples were directly injected onto the GC/TOF system using Method #1. A second intermediate solution of Preblend 1A at 5 mg/mL was prepared by diluting approximately 20 mg of the preblend with dimethylformamide (DMF) containing internal standard (benzene-d_6_ at 1 µg/mL). Sample was further diluted 100-fold in phosphate-buffered saline prior HS injection, Method #2. For Preblends 1C, 2, 3, and 4, an intermediate solution was made by performing a 10-fold dilution, by volume, with DCM. From the intermediate solution, two additional dilutions (10- and 100-fold) were performed using DCM and spiked with internal standard (10 mg tricosane-d_48_ per mL DCM) to a desired concentration of 40 µg/mL. Samples were directly injected onto the GC/MS, Method #3.

### Analytical characterization of test formulations

For the analysis of ingredients from Preblends 1A and 1B, 20 mg of the test formulations was diluted to 20 mg/mL in DCM/MeOH (80/20 v/v) containing internal standard (0.2 µg/mL isophorone-d_8_ per; 1 µg/mL 2-methoxy-4-methylphenol-d_3_, 2,5-dimethyl-d_6_-pyrazine, eugenol-d_3_-acetate, isoamyl alcohol-d_9_, and benzene-d_6_; 2 µg/mL 2-methylbutyric acid-3,4-^13^C_2_ and furaneol-^13^C_2_). This solution was further diluted for injection at up to 200-fold in DCM/MeOH (80/20 v/v) to achieve an appropriate analyte concentration within the linear range of the instrument, Method #1. Test formulations were also diluted to 20 mg/mL in DMF containing internal standard (benzene-d_6_ at 1 µg/mL) prior being further diluted in PBS between 50- and 200-fold and injected in HS mode, Method #2. For the analysis of ingredients from Preblends 1C, 2, 3 and 4, the test formulations were diluted (up to 200-fold) using DCM and spiked with internal standard (10 mg tricosane-d_48_ per mL DCM) to a desired concentration (e.g. 20–1000 µg/mL), Method #3. The analytical testing for preblends and test formulations was performed in accordance with ISO 17 025 principles and demonstrated general fit-for-purpose [29].

### Quantitation of flavor ingredients

The response for each compound was determined using the peak area ratios between the compound of interest and the internal standard. The concentration of the compounds was calculated using an external calibration curve. All instrument performance was evaluated using quality control samples and by performing a system suitability test prior to analysis. Changes in observed levels were expressed as a percentage of the initial value (*T*_0_). An additional set of preblend samples were freshly prepared at the end of the stability assessment and were analyzed the same day as the last time point to confirm any observed variation was due to instrumental variation. Additional investigation for unknown compound identification was done in GC-MS full scan mode.

## Results and discussion

### Preblend analytical characterization

Preblend characterization of the flavor ingredients under refrigerated conditions for all six groups (i.e. Preblends 1A, 1B, 1C, 2, 3, and 4) are presented in Table 2. Preblend Group 5 was not represented since it contained only one compound (2-methylbutyric acid).

**Table 2.** Stability preblends over 4 weeks under refrigerated conditions: percent changes compared to initial (*T*_0_)

	Time point (weeks)				
Compound	Initial	1	2	3	4
**Preblend 1a**					
Paracymene	100%	100%	104%	109%	88%
1-Penten-3-one	100%	98%	101%	103%	89%
Isopulegol	100%	104%	103%	108%	99%
Isobutyraldehyde	100%	95%	92%	96%	113%
d-l-citronellol	100%	105%	105%	102%	109%
Ethyl lactate	100%	106%	103%	105%	113%
*cis*-3-Hexenol	100%	106%	104%	115%	114%
Acetal	100%	103%	98%	103%	114%
2-Methyl-4-phenyl-2-butanol	100%	107%	105%	110%	103%
**Preblend 1b**					
Ambrox (Cetalox^©^)	100%	102%	96%	95%	88%
*trans*-α-Damascone	100%	103%	111%	108%	102%
Linalool	100%	110%	125%	130%	118%
*para*-Dimethoxybenzene	100%	104%	102%	110%	97%
Ethyl 2-methylbutyrate	100%	103%	101%	109%	109%
Acetanisole	100%	104%	102%	103%	91%
Eugenyl acetate	100%	103%	99%	98%	87%
**Preblend 1c**					
Alpha-pinene	100%	101%	101%	97%	95%
Isoamyl alcohol	100%	100%	100%	98%	97%
Benzyl alcohol	100%	101%	102%	100%	98%
2-Methoxy-4-methylphenol	100%	102%	101%	99%	94%
Delta-nonalactone	100%	100%	97%	95%	95%
Ethyl vanillin	100%	96%	97%	92%	89%
**Preblend 2**					
(E, Z)-2,6-Nonadienal	100%	75%	74%	74%	81%
Ketoisophorone	100%	94%	88%	82%	82%
Piperitone	100%	93%	87%	81%	82%
Ethyl maltol	100%	95%	88%	61%	102%
Furaneol	100%	97%	94%	76%	108%
Methyl cinnamate	100%	97%	91%	84%	85%
Dihydroactinidiolide	100%	92%	83%	82%	90%
**Preblend 3**					
3-Methyl-2,4-nonanedione	100%	99%	103%	97%	100%
Triethyl citrate	100%	96%	106%	97%	90%
**Preblend 4**					
2,5-Dimethylpyrazine	100%	99%	98%	87%	100%
3-Ethylpyridine	100%	100%	98%	89%	100%
2-Acetylthiazole	100%	98%	94%	85%	95%
*para*-Mentha-8-thiol-3-one (Isomer 1)	100%	101%	98%	84%	98%
*para*-Mentha-8-thiol-3-one (Isomer 2)	100%	101%	98%	83%	97%
2-Acetylpyrrole	100%	99%	95%	85%	97%
Methyl anthranilate	100%	100%	98%	89%	99%

Preblend 1A contained a mixture of highly volatile compounds such as aldehydes, alcohols, acetals, and ketones. All flavor ingredients were within ±15% of the initial measured concentration over the 4-week assessment period with no notable trends and no newly formed peaks were observed in the TIC from the GC-MS full scan. Preblend 1B also showed all flavor ingredients within ±15% of the initial measured concentration over the 4-week assessment. One exception was linalool which appeared to increase over time; however, when this solution was compared to a freshly made solution of Preblend 1B, the results were within 10% which indicated instrument variability and not stability. Although there was variability in the quantitation, it did not impact the overall results of the study and there was no indication of degradation. All flavor ingredients in Preblend 1C were within ±15% of the initial measured concentration over the 4-week assessment.

The concentration of ethyl vanillin in Preblend 1C stored at room temperature dropped below 85% of the initial concentration compared to *T*_0_ and levels continued to decline over the 4 week of analysis (Supplemental Data, Fig. 1). A downward trend was observed for refrigerated storage temperature as well but not as dramatic. During the stability study period, a new peak was observed in the TIC GC-MS analysis. Additional analysis was conducted to characterize this newly formed peak to determine the molecular weight and formula. The investigation found that ethyl vanillin was reacting with PG to form its PG acetal analogues. The formation of PG acetals, specifically from reactions with flavor aldehydes, has been reported in other e-vapor formulations [30]. For instance, Erythropel *et al*. reported that acetal formation within a PG matrix is driven by several factors, which include (acidic) pH, high PG/VG ratio, water content, and competition with other reactants or flavors. In Preblend 1C, some of the aforementioned factors may have aided in the formation of ethyl vanillin PG acetal.

Preblend 2 contained electrophilic α, β-unsaturated ketones and aldehydes. Most of the flavor ingredients were within ±15% of the initial measured concentration over the 4-week assessment (Table 2). Compared to other preblend groups, this group of flavors showed greater variation in Weeks 2–4. In particular, (E, Z)-2,6-nonadienal showed a trend, in which the initial measured concentration decreased rapidly in the first week to approximately 75% of the initial concentration. Similar to the ethyl vanillin investigation, the formation of an acetal was observed; however, the conversion was complete within hours of preblend preparation. Unlike the ethyl vanillin PG acetal, (E, Z)-2,6-nonadienal PG acetals had a relatively stable equilibrium from Weeks 1 to Week 4. Since the formation of the acetal resulted in a stable form and the formation was unavoidable in PG, it was determined to be most representative of what would be expected in a flavor solution. Additionally, for Preblend 2 at Week 3, significant peak tailing was observed for ethyl maltol and furaneol, chromatographic issues have been reported with these compounds in a PG/VG matrix [31]. Following column maintenance, including cutting the front end of the column to remove any build-up, Week 4 data point was within ±15% of their initial measured concentrations. Ketoisophorone and piperitone showed a trend in decreasing concentration over Week 3 and remained below the 85% acceptance limit at Week 4. It is recommended to limit the stability of this preblend to Week 4 to ensure the levels of for these analytes in the test formulation are within 20% of the target concentration.

Preblend 3 contained only two ingredients, 3-methyl-2,4-nonanedione and triethyl citrate, both of which are enolizable nucleophilic β-dicarbonyls (Table 1). Both ingredients were stable within ±15% of their initial measured concentrations over the 4-week assessment. No notable trends or newly formed peaks were observed (Table 2).

Preblend 4 contained basic compounds containing nitrogen and sulphur. All flavor ingredients were stable within ±15% of their initial measured concentrations over the 4-week assessment. There was some variability observed with the quantitation of these compounds most notably at Week 3; however, the results for Week 4 were all within the acceptance criteria. Overall, no notable trends or newly formed peaks were observed (Table 2).

### Characterization of test formulation under room-temperature conditions

In *in vivo* inhalation studies, a 6-h daily exposure is often the maximum exposure period [32]. To ensure that the test formulations were stable during the exposure period (including preparation time), test formulations were analyzed for flavor ingredients at room temperature over 3 days. Tables 3 and 4 show the change in concentration of the 38 flavor ingredients in the test formulations with and without nicotine, respectively. The test formulation without nicotine was observed to be more stable than the test formulation with nicotine as evidenced by (E, Z)-2,6-nonadienal decreasing by approximately 20% over 3 days. One p-mentha-8-thiol-3-one isomer also decreased over 3 days; however, the sum of the two isomers was stable for 3 days. Overall, the results indicated that all 38 flavor ingredients assessed were within ±20% of the initial measured concentration and considered acceptable for up to 3 days at room temperature, which was sufficient for the daily exposure study.

**Table 3.** Stability results of high test formulation containing all 38 compounds with nicotine—room temperature (RT) and refrigerated (REF) conditions

	*T*_0_	1 Day RT	1 Day REF	3 Days RT	3 Days REF	7 Days REF	11 Days REF
**Group 1A**							
Paracymene	100%	104%	97%	108%	NA	96%	97%
1-Penten-3-one	100%	99%	93%	97%	NA	56%	45%
Isopulegol	100%	95%	95%	104%	NA	93%	94%
Isobutyraldehyde	100%	NT	88%	101%	NA	84%	91%
d-l-citronellol	100%	101%	96%	99%	NA	90%	91%
Ethyl lactate	100%	105%	96%	123%	NA	90%	94%
*cis*-3-Hexenol	100%	103%	97%	108%	NA	96%	93%
Acetal	100%	104%	111%	110%	NA	106%	107%
2-Methyl-4-phenyl-2-butanol	100%	99%	97%	102%	NA	98%	97%
**Group 1B**							
Ambrox (Cetalox^©^)	100%	105%	98%	100%	NA	95%	94%
*trans*-α-Damascone	100%	100%	96%	110%	NA	90%	89%
Linalool	100%	99%	90%	104%	NA	83%	81%
*para*-Dimethoxybenzene	100%	106%	96%	110%	NA	96%	94%
Ethyl 2-methylbutyrate	100%	104%	107%	107%	NA	106%	114%
Acetanisole	100%	105%	94%	115%	NA	92%	89%
Eugenyl acetate	100%	104%	98%	108%	NA	97%	95%
**Group 1C**							
Alpha-pinene	100%	102%	103%	110%	102%	109%	105%
Isoamyl alcohol	100%	100%	101%	108%	99%	104%	104%
Benzyl alcohol	100%	100%	101%	107%	99%	104%	105%
2-Methoxy-4-methylphenol	100%	100%	101%	108%	100%	107%	106%
Delta-nonalactone	100%	96%	99%	98%	95%	99%	99%
Ethyl vanillin	100%	100%	101%	108%	101%	106%	107%
**Group 2**							
(E, Z)-2,6-Nonadienal	100%	90%	94%	79%	89%	89%	79%
Ketoisophorone	100%	99%	100%	105%	98%	104%	104%
Piperitone	100%	99%	100%	107%	98%	106%	106%
Ethyl maltol	100%	101%	100%	104%	104%	111%	106%
Furaneol	100%	94%	96%	83%	90%	93%	86%
Methyl cinnamate	100%	100%	101%	107%	100%	107%	106%
Dihydroactinidiolide	100%	99%	101%	106%	98%	106%	106%
**Group 3**							
3-Methyl-2,4-nonanedione	100%	101%	102%	107%	102%	105%	104%
Triethyl citrate	100%	102%	103%	109%	103%	109%	110%
**Group 4**							
2,5-Dimethylpyrazine	100%	100%	101%	108%	99%	106%	105%
3-Ethylpyridine	100%	100%	101%	107%	99%	106%	105%
2-Acetylthiazole	100%	100%	101%	107%	100%	108%	105%
*para*-Mentha-8-thiol-3-one, Isomer 1	100%	71%	79%	72%	63%	53%	49%
*para*-Mentha-8-thiol-3-one, Isomer 2	100%	95%	98%	94%	94%	93%	91%
*para*-Mentha-8-thiol-3-one, Sum	100%	83%	88%	83%	78%	73%	70%
2-Acetylpyrrole	100%	100%	102%	108%	100%	106%	106%
Methyl anthranilate	100%	93%	98%	92%	94%	96%	92%
**Group 5**							
2-Methylbutyric acid	100%	100%	99%	99%	99%	107%	100%
**Additional compound**							
Nicotine	100%	98%	97%	100%	99%	115%	102%

NT, not tested: analysis of isobutyraldehyde was not performed on Day 1 under room temperature conditions.

NA, not analyzed: data for 3 days of refrigerated storage were not collected due to laboratory constraints during the experimental setup.

Acceptance criteria for test formulation = ±20% of initial value (*T*_0_).

**Table 4.** Stability results of high test formulation containing all 38 compounds without nicotine—room temperature (RT) and refrigerated (REF)

	*T*_0_	1 Day RT	1 Day REF	3 Days RT	3 Days REF	7 Days REF	11 Days REF
**Group 1A**							
Paracymene	100%	98%	102%	100%	NA	104%	94%
1-Penten-3-one	100%	103%	99%	94%	NA	92%	81%
Isopulegol	100%	102%	103%	106%	NA	104%	88%
Isobutyraldehyde	100%	NT	106%	103%	NA	102%	86%
d-l-citronellol	100%	102%	100%	99%	NA	91%	82%
Ethyl lactate	100%	104%	95%	128%	NA	98%	92%
*cis*-3-Hexenol	100%	97%	99%	100%	NA	101%	87%
Acetal	100%	100%	102%	106%	NA	107%	95%
2-Methyl-4-phenyl-2-butanol	100%	104%	99%	105%	NA	99%	88%
**Group 1B**							
Ambrox (Cetalox^©^)	100%	104%	99%	105%	NA	96%	95%
*trans*-α-Damascone	100%	101%	101%	103%	NA	96%	95%
Linalool	100%	100%	93%	97%	NA	90%	86%
*para*-Dimethoxybenzene	100%	103%	96%	103%	NA	93%	92%
Ethyl 2-methylbutyrate	100%	99%	100%	102%	NA	104%	105%
Acetanisole	100%	101%	95%	104%	NA	90%	89%
Eugenyl acetate	100%	102%	97%	105%	NA	95%	95%
**Group 1C**							
Alpha-pinene	100%	99%	101%	105%	105%	103%	100%
Isoamyl alcohol	100%	97%	98%	104%	104%	99%	98%
Benzyl alcohol	100%	96%	97%	102%	101%	101%	97%
2-Methoxy-4-methylphenol	100%	97%	98%	103%	102%	103%	98%
Delta-nonalactone	100%	96%	96%	98%	99%	102%	96%
Ethyl vanillin	100%	98%	98%	102%	103%	105%	100%
**Group 2**							
(E, Z)-2,6-Nonadienal	100%	98%	98%	103%	103%	99%	92%
Ketoisophorone	100%	95%	97%	101%	101%	101%	97%
Piperitone	100%	96%	97%	101%	100%	102%	97%
Ethyl maltol	100%	102%	102%	106%	106%	110%	104%
Furaneol	100%	97%	97%	94%	94%	101%	96%
Methyl cinnamate	100%	96%	97%	102%	102%	103%	98%
Dihydroactinidiolide	100%	96%	96%	101%	100%	105%	97%
**Group 3**							
3-Methyl-2,4-nonanedione	100%	100%	100%	105%	106%	105%	101%
Triethyl citrate	100%	102%	102%	108%	107%	114%	106%
**Group 4**							
2,5-Dimethylpyrazine	100%	96%	97%	103%	102%	101%	97%
3-Ethylpyridine	100%	96%	98%	107%	107%	102%	98%
2-Acetylthiazole	100%	95%	98%	105%	103%	101%	97%
*para*-Mentha-8-thiol-3-one, Isomer 1	100%	93%	99%	92%	100%	96%	88%
*para*-Mentha-8-thiol-3-one, Isomer 2	100%	97%	98%	103%	103%	102%	96%
2-Acetylpyrrole	100%	97%	98%	104%	102%	103%	98%
Methyl anthranilate	100%	94%	97%	96%	99%	98%	92%
**Group 5**							
2-Methylbutyric acid	100%	98%	98%	102%	102%	105%	97%

NT, not tested: analysis of isobutyraldehyde was not performed on Day 1 under room temperature conditions.

NA, not analyzed: data for 3 days of refrigerated storage were not collected due to laboratory constraints during the experimental setup.

Acceptance criteria for test formulation = ±20% of initial value (*T*_0_).

### Characterization of test formulation under refrigerated conditions

Stability of test formulations under refrigerated conditions was evaluated to establish the preparation schedule. Same day test formulation, characterization, and exposure are challenging, but if the test formulation is stable under refrigerated conditions, then preparation can occur prior to the day of *in vivo* exposure. Tables 3 and 4 show the change in concentration of the 38 flavor ingredients in the test formulations with and without nicotine, respectively, over 11 ± 1 days at refrigerated storage conditions (4°C ± 2°C). It is noted that, due to laboratory resource constraints, compounds from Group 1a and Group 1b were not analyzed on Day 3. In general, 35 of the 38 flavor ingredients, regardless of the presence of nicotine, remained unchanged over the course of the refrigerated storage period. For the test formulation without nicotine, all 38 flavor ingredients were within ±20% of the initial measured concentration and, therefore, acceptable for use in subsequent inhalation exposures up to 10 days. The test formulation with nicotine showed three flavor ingredients that fell below 80% of the initial measured concentration at the 7-day time point. Data from the Day 3 room temperature and refrigerated data were used to support the use of these test solutions for up to 3 days when stored under refrigerated conditions (Table 3).

The flavor ingredients that displayed a downward trend in measured concentration warranted further investigation. We conducted a Non-Targeted Analysis (NTA) investigation to investigate the instability of 1-penten-3-one. In literature, 1-penten-3-one is a well-studied aroma compound commonly associated with grapefruit, orange juice, black tea, olive oil, or tomatoes [33, 34]. Mall V, *et al*., studied the refrigerated stability of 1-penten-3-one in freshly squeezed orange juice and determined that its unstable nature was in part due to a condensation reaction with l-cysteine. In our study, NTA analysis showed that 1-penten-3-one reacted with methyl anthranilate and p-menth-8-thiol-3-one to form the resulting condensation reaction products, which was consistent with its reactivity observed by Mall V, *et al*. Further, the condensation reactions of 1-penten-3-one with both p-mentha-8-thiol-3-one and methyl anthranilate were also supported by the decrease from their initial concentrations over the 11-day storage period (Table 2). We however caution that there is a possibility of additional reactions that could not be accounted for due to chromatographic separation or limited method sensitivity. For (E, Z)-2,6-nonadienal, although no additional experiments were performed, we speculate that upon mixing of Preblend 2, the equilibrium between the flavor ingredient and resulting PG acetal(s) was disrupted by the addition of water, VG, and additional flavor ingredients [30].

## Conclusion

In this study, we demonstrated a pragmatic approach of grouping 38 flavor ingredients into five concentrated preblends, simplifying the timely preparation of test formulations needed for day-to-day operations in *in vivo* inhalation studies. Grouping the compounds based on potential reactivity minimized the chemical reactions and provided acceptable stability for the preblends when stored under refrigerated conditions. For all the tested flavor ingredients, we were able to demonstrate acceptable stability of the test formulation for up to 3 days under room temperature and refrigerated conditions, suitable for 6-h daily exposure studies, and allow the test formulation to be prepared in advance and used for multiple days. While this approach successfully minimized chemical reactions between flavors compounds, it is important to note some of the limitations. For example, some chemical reactions among e-vapor ingredients are inevitable, for instance, interactions between certain flavors and the common carrier ingredient, PG. The formation of PG acetals has been previously studied in e-formulations [30], and in this study, we also observed for ethyl vanillin and (E, Z)-2,6-nonadienal. This suggests that the formation of the PG acetals cannot be avoided and likely represents many e-liquid formulations in the market. In addition, the stability of e-liquid formulations can be impacted by many factors including pH and nicotine form and should be fully characterized prior to toxicity testing. At the same time, the study also highlights the challenges of characterizing test articles of complex mixtures prior to toxicity studies.

In conclusion, we demonstrate that the use of preblends can enhance the assurance of predictable stability of test formulations when there is a need to evaluate a large number of compounds and substantially simplifies the preparation of test materials used for long-term toxicological assessment.

## Data Availability

The data underlying this article will be shared on reasonable request to the corresponding author.
